# Supplementary material for: A revision of the rare Strumigenys mnemosyne (Formicidae; Myrmicinae) group using micro-CT scanning, with the description of three new species, and the virtual repair of a broken paratype
Source: PLoS One. 2025 Sep 24;20(9):e0331762. doi: 10.1371/journal.pone.0331762 (PMC12459837; doi:10.1371/journal.pone.0331762)
Supplement: Attachment — Submitted filename: PONE-D-25-13901 Reviewing conclusion 20250607 Zhenghui Xu.pdf [file pone.0331762.s001.pdf]

PONE-D-25-13901 Reviewing conclusion 20250607 by Zhenghui Xu

After review of the manuscript PONE-D-25-13901, I believe the three species described, *S. liui* sp. nov., S. *marmorata* sp. nov., and *S. rimdahli* sp. nov., are new to science. The discovery of new members obviously increased our knowledge of the rare *Strumigenys mnemosyne* group. The results are worth to publish on the journal. However, there are numerous errors or inappropriate expressions in the manuscript. It needs to be published after major revisions.

1. Line 6: Change "Kadoorie Biological Sciences, Building" to "Kadoorie Biological Sciences Building".
2. Lines 60-61: Change "singleton holotype specimens" to "singleton holotype specimen".
3. Lines 89-91: This sentence "We provide an updated dichotomous key and distributional checklist and map of the whole S. mnenosyne group, and high-resolution images of all species, including the first for *S. runa* (Bolton, 2000)" used too many "and", and the meaning of last part is not clear. I suggest the authors to use simple sentences to express clearly.
4. Lines 110-135: The authors used some complex abbreviation for the measurements "PrW, PetL, PetH, DPetW, DPetL, DPPetW, DDPetL", why don't you use the simple abbreviation "PW, PL, PH, DPW, DPL, PPW, PPL" as in Bolton (2000)? Because scientific report is trying to make thing simple, not the contrary.
5. Lines 157-179: The authors habitually used too many "we" in the whole paragraph and seems too colloquial. I suggest the authors change the expression of sentences and reduced the use of "we" frequently.
6. Lines 184-185: "Hita Garcia and colleagues (2025)" is not a standard literature citation, please change it to "Hita Garcia et al. (2025)".
7. Line 240: This sentence "Dorsum and sides of the mesosoma declivity of propodeum" seems incorrect, please rewrite.
8. Line 262: Change "1 Holotype worker" to "Holotype worker", because holotype is a single individual.
9. Lines 269-270: In "Nothing is known of the biology of *S. daspleta* but it is presumed to have been collected within leaf litter", the subjective guess "but it is presumed to have been collected within leaf litter" is perilous and maybe cause a mistake. If the biological information of the species was not recorded in the original description, it is better keeping unknown until new information obtained in the future investigation.
10. Line 275: Table 1 short of an end line.
11. Line 289: Change "Physical specimens examined (n=1)" to "Physical specimen examined (n=1)".
12. Line 290: Change "1 Holotype worker" to "Holotype worker"; change "Baihua Lin" to "Baihua Ling".
13. Line 290: Change "N25.302, E.098.789" to "N25.302, E98.789".
14. Line 302: Change "Holotype worker (n = 1)" to "Holotype worker", "(n = 1)" is redundant.
15. Line 306: Change "Occiput" to "Posterior margin", the correct name of this part is posterior margin in morphology.
16. Line 310: Change "in full face view" to "in full-face view".
17. Line 314: Change "upper masticatory margin" to "Basal masticatory margin".
18. Lines 314-315: Change "lower half" to "apical half".
19. Line 319: Change "convex below eye" to "convex before eye".
20. Lines 323-324: The meaning of "Mesosoma undifferentiated" is not clear. It is better to change the whole sentence "Mesosoma undifferentiated, lacking pleural sutures other than weakly impressed promesonotal suture not reaching dorsolateral margin in lateral view" to "Mesosoma lacking

promesonotal suture on the dorsum, metanotal groove absent".

21. Line 326: What is "Hair wheel"?
22. Line 334: This description "Petiolar spiracle located just below node on anterolateral region of peduncle" is wrong, please check and rewrite.
23. Line 341: About pilosity, the setae in Fig.3D-F are obviously shorter and stouter than in Fig.3A-C, why? It seems the shape of setae in Fig.3D-F distortion. Similar in Fig.4-8.
24. Line 346: Change "apically pointed setae" to "apically acute setae".
25. Line 354: It is not clear whose "Tergite one"? please rewrite.
26. Line 359: Change "Mandibles and antennal segments dulled by dense microsculpture" to "Mandibles and antennal segments with dense micropuncture". Sculpture is a general term for all kind of sculptures.
27. Line 362: Change "Overall body reddish-orange, appendages yellow. Spongiform tissue yellow-white" to "Body reddish orange, appendages yellow. Spongiform tissue light yellow".
28. Line 385: Change "tolerance for higher elevations conditions" to "tolerance for higher elevation condition".
29. Line 395: Change "In full face view" to "In full-face view".
30. Lines 395-396: "in lateral view" is repeated.
31. Line 397: Change "Upper masticatory margin" to "Basal masticatory margin".
32. Line 398: Change "lower half" to "apical half".
33. Line 406: Change "1 Holotype worker" to "Holotype worker".
34. Line 406: Change "22.26906 113.93088" to "N22.26906, E113.93088".
35. Line 409: Change "22.51211 114.0928" to "N 22.51211, E114.0928".
36. Line 428: Change "Holotype measurements (n = 1)" to "Holotype measurements".
37. Line 435" Change "Occiput" to "Posterior margin".
38. Line 438: Change "in full face view" to "in full-face view".
39. Line 439: Change "occiput" to "posterior margin".
40. Lines 441-442: Change "upper masticatory margin" to "basal masticatory margin", change "lower half" to "apical half".
41. Line 445: Change "single ommatidia" to "single ommatidium".
42. Line 446: Change "convex below eye" to "convex before eye".
43. Line 450: The meaning of "Mesosoma undifferentiated" is not clear. It is better to change the whole sentence "Mesosoma undifferentiated, lacking pleural sutures other than weakly impressed promesonotal suture not reaching dorsolateral margin in lateral view" to "Mesosoma lacking promesonotal suture on the dorsum, metanotal groove absent".
44. Line 459-460: Anterior peduncle of petiole is much longer in Fig. 4A than in Fig. 4E, they are the same species, why? Meanwhile, anterior margin of postpetiole weakly concave in Fig.4C, in contrast, anterior margin of postpetiole straight in Fig.4F, why? Are they really belonging to the same species?
45. Line 462: "kidney bean shaped" is not a good description, please describe using geometry terms.
46. Line 462: Change "postpetiolar disc conspicuously concave" to "postpetiole conspicuously concave".
47. Line 463: "parabolic arc" is not accurate, please rewrite.
48. Lines 481-482: Change "post-petiole" to "postpetiole", change "onepair" to "one pair".
49. Line 486: Change "meso- and metabasitarsi and tibiae" to "meso- and meta- basitarsi and tibiae".
50. Lines 490-492: Change "Core body segments reddish-brown; appendages of similar but lighter colour; antennal segments brighter compared to body, considerably more orange; spongiform tissue of typical whitish yellow colouration" to "Body color reddish brown, appendages light reddish brown, spongiform tissue light yellow".

51. Line 495: Change “micro-sculpture” to “micro-punctures”.
52. Lines 508-522: In this paragraph, the authors say “*S. marmorata* most closely resembles *S. mnemosyne* and *S. rimdahli*”. They have compared *S. marmorata* and *S. mnemosyne* carefully, but no difference mentioned between *S. marmorata* and *S. rimdahli*. Please supplement and improve.
53. Lines 528-529: Change “*Strumigenys emmae,* (Emery, 1890)” to “S*trumigenys emmae* (Emery, 1890)”.
54. Line 537: Change “Digital images of holotype (A) lateral, (B) dorsal, (C) full face view” to “Digital images of holotype (A) head, (B) lateral, (C) dorsal”.
55. Line 556: Change “1 Holotype worker” to “Holotype worker”.
56. Line 560: Change “22.42007 114.1829” to “N22.42007, E114.1829”, change the similar latitude and longitude in the whole paragraph from Line 558 to Line 580, and on Lines 603-610.
57. Line 622: Indices of gyne: CI 75.91–131.18, which showing a huge span and seems unusual, please check and make sure.
58. Line 625: Change “occipital border” to “posterior border”.
59. Lines 626-627: Change “upper masticatory margin” to “basal masticatory margin”, change “lower half” to “apical half.
60. Line 636: Change “Occipital border” to “Posterior margin”.
61. Lines 641: Change “mesoscutum anterior margin” to “anterior margin of mesoscutum”.
62. Line 655: The word “subclubate” seems wrong, please check and correct.
63. Line 659: Change “ventral petiole” to “ventral face of petiole”.
64. Lines 659-660: Change “First gastralastral tergite long, as long as petiole and postpetiole” to “First gastral tergite long, as long as petiole and postpetiole combined”.
65. Lines 680-688: Please make sure what is “microsculpture”, micro-puncture or micro-reticulation? Because “sculpture” is a general name for every kind of sculpture including puncture, reticulation, etc.
66. Line 690: Change “Overall body and appendages red-orange, spongiform tissue yellow-white” to “Body and appendages reddish orange, spongiform tissue light yellow”.
67. Line 726: Change “1 Holotype worker” to “Holotype worker”.
68. Line 753: Change “1 Holotype worker” to “Holotype worker”, change “19.236638 98.650739” to “N19.236638, E98.650739”.
69. Line 769: Change “Occiput” to “Posterior margin”.
70. Line 773: Change “occiput” to “posterior margin”.
71. Lines 775-776: Change “upper masticatory margin” to “basal masticatory margin”, change “lower half” to “apical half”.
72. Line 780: Change “single ommatidia” to “single ommatidium”.
73. Line 781: Change “below eye” to “before eye”.
74. Lines 785-786: The meaning of “Mesosoma undifferentiated” is not clear. It is better to change the whole sentence “Mesosoma undifferentiated, lacking pleural sutures other than weakly impressed promesonotal suture not reaching dorsolateral margin in lateral view” to “Mesosoma lacking promesonotal suture on the dorsum, metanotal groove absent”.
75. Lines 880 and 802: Change “post-petiole” to “postpetiole”.
76. Line 815: Change “fourc to five” to “four to five”.
77. Lines 824-826: Change “Core body segments reddish-brown; appendages of similar but lighter colour; antennal segments brighter compared to body, considerably more orange; spongiform tissue of typical whitish yellow colouration” to “Body color reddish brown, appendages light reddish brown, spongiform tissue whitish yellow”.

78. Line 829: Please make sure what kind of sculpture on antennae, coxae and basitarsi.
79. Line 838: Change “rapidly” to “easily”.
80. Line 843: Chang “in *mnemosyne*” to “in *S. mnemosyne*”.
81. Line 852: Change “of the *Strumigenys rimdahli* sp. nov.” to “of *Strumigenys rimdahli* sp. nov.”.
82. Line 853: Change “Digital images (A) lateral view, (B) dorsal view, (C) full face view” to “Digital images (A) head, (B) lateral, (C) dorsal”.
83. Line 865: Change “4.73356 116.87757 ± 50m” to “N4.73356, E116.87757”.
84. Line 875: Change “Digital images (A) lateral view, (B) dorsal view, (C) full face view” to “Digital images (A) head, (B) lateral, (C) dorsal”, meanwhile, rearrange the images of Fig.9 in the order: (A) head, (B) lateral, (C) dorsal.
85. Line 878” Change “Fig 2G–I” to “Fig. 2G–I”.
86. Line 884: Change “highly concave” to “deeply concave”.
87. Line 889: Change “1 Holotype worker” to “Holotype worker".
88. Lines 907-908: Change “Key to the *Strumigenys mnemosyne* group (adapted from Bolton 2000; Southeast Asia *Strumigenys* key)” to “Key to species of the *Strumigenys mnemosyne* group based on worker caste”.
89. Line 909: Change “This key branches from couplet 84 of Bolton (2000), ending at couplet 87” to “(Adapted from the Southeast Asia Strumigenys key of Bolton, 2000, branching from couplet 84 and ending at couplet 87)”.
90. Line 911: Because the opposite sentence of antithetical couplet 1 short of “Basigastral costulae” character, please delete “Basigastral costulae absent” here.
91. Line 917: Change “anterodorsally . . . . . 4” to “anterodorsally . . . . . 5”.
92. Lines 935-939: It seems the characters the authors provided to separate the two species are hard to use. Please find more other clear characters to separate the two species.
93. Line 950: Change “The mnemosyne group” to the mnemosyne group
94. Line 952: Change “absence of spines” to “absence of propodeal spines”.
95. Line 954: Change “the mnemosyne group” to “the *S. mnemosyne* group”.
