# Supplementary material for: A revision of the rare Strumigenys mnemosyne (Formicidae; Myrmicinae) group using micro-CT scanning, with the description of three new species, and the virtual repair of a broken paratype
Source: PLoS One. 2025 Sep 24;20(9):e0331762. doi: 10.1371/journal.pone.0331762 (PMC12459837; doi:10.1371/journal.pone.0331762)
Supplement: Attachment — Submitted filename: Response to Reviewers.docx [file pone.0331762.s004.docx]

Response to the editor

We would like to thank the editor, and the three reviewers for their thoughts and comments on our manuscript. Nearly all comments from reviewer 2 and 3 have been included into the manuscript. Where their comments have not been included in the manuscript, a rebuttal has been provided. Concerning concerns raised by reviewer 1, their comments are based more in philosophy, are somewhat irrelevant to our paper, and based on unscientific presumptions which are substantiated by the current literature. Further, they seem to be confused by the aims and scope of PLOS One, suggesting instead that we should submit to a taxonomy specific journal, but provide no justification for this. Therefore, we believe that their comments are mostly irrelevant to our paper.

Our responses to the reviewers can be found in red.

Reviewer 1 response

**Reviewer #1:** I appreciate the opportunity to review this manuscript and would like to extend my gratitude to both the authors and the editorial team for their efforts. The current research presents a taxonomic reassessment of the mnemosyne species group of the myrmicine genus Strumigenys, providing descriptions of three new species, commentary on previously described species, an identification key for all species within the group, and a workflow for the “virtual reconstruction” of broken specimens using micro-CT models.

This study represents a well-executed taxonomic analysis and will be a valuable addition to the Strumigenys literature. However, I believe it requires significant revisions to be suitable for publication in a taxonomy-oriented journal. More critically, I do not see how this study advances the broader fields of Biology or Taxonomy, as it presents two major issues that hinder its suitability for publication in this journal.

First, the authors argue that micro-CT models can help mitigate the risk of specimen breakage during handling and provide a method for reconstructing damaged specimens. However, this justification is problematic, as the specimen needing reconstruction was damaged during the micro-CT process itself. In this context, the “reconstruction workflow” appears to be a solution to a problem introduced by the study’s own methodology. Additionally, contrary to the authors’ claims, the micro-CT models did not contribute to the observation of difficult-to-see structures in the head, such as the dentition formula. These structures are more clearly visible in the high-resolution images, as evidenced by the difficulty in distinguishing smaller preapical teeth in the micro-CT model of S. marmorata compared to the corresponding high-resolution image.

Second, while I partly agree with the authors that digital surrogates could help alleviate problems associated with operational procedures (e.g. breaking specimens), it is important to highlight that it does not alleviate the issue of accessibility to physical type specimens housed in collections when intended to be used for revisionary studies, nor solves the issues of reproducibility or replicability in taxonomy. Since "digital objects do not represent anything, in either sense, but gain roles and capacities in their use in different social settings” (Boast & Enote, 2013), cybertypes would serve only as iconic representational artifacts (i.e. human-made concretizations of cognitive representations that carry aesthetic nonconceptual content by resemblance to the physical item), and, thus, would play the same role as 2D images. This means that they could not be used by other researchers interested in reevaluating species boundaries, because data (in this case, formalized statements of morphological features made by a researcher based on the physical specimen) would have to be created by documenting morphological features observed directly from the physical type specimen; otherwise, researchers would be only describing human-made concretizations, instead of material entities (like describing Dorian Grey based on Henrique Medina’s painting).
Given these concerns, I recommend rejecting this manuscript for publication in this journal and suggest that the authors consider submitting it to a taxonomy-focused journal instead.
Thank you once again for the opportunity to review this manuscript.

Boast, R., Enote, J. (2013). Virtual Repatriation: It Is Neither Virtual nor Repatriation. In: Heritage in the Context of Globalization. SpringerBriefs in Archaeology, vol 8. Springer, New York, NY. https://doi.org/10.1007/978-1-4614-6077-0_13

Response: We would like to thank reviewer 1 for their thoughts on our manuscript, however we think the arguments presented are somewhat irrelevant here, overly philosophical, and do not reflect the scientific content presented in our study. Below we provide a rebuttal to the ideas and opinions they have presented.

Firstly, from our understanding, it seems that the reviewer is confused by the aims and scope of PLOS One. Reviewer 1 has stated ‘*I do not see how this study advances the broader fields of Biology or Taxonomy*’. On the PLOS One webpage *Journal Information* (<https://tinyurl.com/yc42c2p3>) it states that ‘… *we evaluate research on the basis of scientific validity, strong methodology, and high ethical standards—not perceived significance*’. As also put by reviewer 1‘*This study represents a well-executed taxonomic analysis and will be a valuable addition to the* Strumigenys *literature*’, this rather contradictory statement implies our paper has met PLOS One standards by being both scientifically valid and methodology in terms of methodology. Despite those statements, the reviewer suggests our paper should be revised and submitted to a taxonomic journal (without reasoning). However, there is no greater generality or novelty required for PLOS ONE, and as inadvertently stated by the reviewer themselves, our paper has met the journals requirements. Nevertheless, we argue below that the points they raise in their review can largely be discarded as those are either the results of a misunderstanding (to which we bring precisions in this new version) or irrelevant to our study.

During further dialogue with coauthors, it was realised that the specimen broke not during the CT-scanning process itself, but upon opening the specimen transportation box before any other manipulation of the specimen. This has been rectified within the manuscript as “*The paratype specimen broke into three pieces whilst opening the specimen transportation box (Fig 5).*”. Despite this, even if the specimen was broken because of our need to conduct the CT-scanning, it does not necessarily mean a problem arises because of the methodology used. Accidents may happen during any protocol, occurring for any reason, and not be fundamentally derived from the methodology itself. In our case, just because our broken specimens were destined for CT scanning (with the potential for virtual repair post scanning), does not equate to CT scanning introducing a problem. It just so happens an accident occurred prior to the application of a method that provided a solution to the problem. A similar accident could have happened for a traditional taxonomic revision for gaining new observations, or placing the specimen in a new collection.

The suggestion that the CT scanning images did not contribute to the observation of difficult to see structures is incorrect. The lead author was not able to discern the full dentition formula in any of the digital images, nor with a microscope at 150x magnification. Access to a 3D model was therefore invaluable in distinguishing the preapical teeth. It is not clear how the reviewer came to their conclusion as they do not have access to digital images of the apical and pre-apical dentition for comparison. Digital images of the pre-apical and apical dentition were not included because of the poor image resolution.

The use of CT scanning for taxonomic purposes has been in use now for over a decade. Virtual models of zoological specimens have enabled the observation of challenging to see morphological structures (Hita Garcia *et al*., 2017; Hita Garcia *et al*., 2019; Hita Garcia *et al*., 2025). Unless a specimen is physically dissected, which is not always possible, even previously hidden internal structures can be observed through virtual dissection (Richter *et al*., 2021; Aibekova *et al*., 2025). From our understanding, the reviewer has argued that morphological characters, observed on 3D models do not represent anything, and are merely human concretions. We believe that this is wrong, with this argument based more in philosophy rather than hard evidence. Numerous previously studies have utilised and incorporated CT scanning models into their character examinations. The reviewer also suggests cybertypes are meaningless given that they represent ‘*nonconceptual content by resemblance to the physical item … and, thus, would play the same role as 2D images’.* The notion that a character does not exist because it is only represented in virtual form is overly philosophical and unsubstantiated. 3D models have all morphological characters as the physical specimen, corresponding directly to the physical specimen rather than simply resembling it (Hita Garcia et al., 2017). Equating a 3D model to a 2D digital image is also highly dubious. With 3D models, one can dissect, rotate and zoom into a model to examine a specimen’s morphology, often allowing to perform measurements which are all impossible for digital images.

Reviewer 1 also states *‘it is important to highlight that it does not alleviate the issue of accessibility to physical type specimens housed in collections when intended to be used for revisionary studies, nor solves the issues of reproducibility or replicability in taxonomy*’. We believe that CT scanned type species do indeed help to alleviate the issue regarding accessibility to type specimens as 3D models provide a powerful and flexible way to visualise specimens, with much greater accessibility than physical specimens as researchers only require a computer connected to the internet. We do not suggest 3D models should replace physical type specimens, nor do we argue this in the paper. But 3D models, surely offer researchers new alternatives and powerful way to access, view, and visualise type material. Further, the reviewer has referenced a single study referring to the repatriation of archaeological and historical artifacts of societal and cultural significance via 3D scans (Boast & Enote 2013), arguing that the artefacts religious and cultural value are not translated via 3D modelling. We believe that this opinion is not applicable to 3D scans of insect specimens, which have low cultural or to any religious value. Further, the issue of repatriation here is irrelevant, considering all physical type specimens and non-type specimens will be deposited within publicly accessible museums in the region where those specimens have been collected.

References

Aibekova L, Richter A, Beutel RG, van de Kamp T, Economo EP, Griebenow Z, Boudinot B. (2025) The Mesosoma of *Protanilla* (Leptanillinae) and the Groundplan of the Formicidae (Hymenoptera), Journal of Morphology, 286(7). https://doi.org/10.1002/jmor.70064.

Boast R, Enote J (2013). Virtual Repatriation: It Is Neither Virtual nor Repatriation. In: Heritage in the Context of Globalization. SpringerBriefs in Archaeology, vol 8. Springer, New York, NY. https://doi.org/10.1007/978-1-4614-6077-0_13

Hita Garcia F, Fischer G, Liu C, Audisio TL, Economo EP (2017) Next-generation morphological character discovery and evaluation: an X-ray micro-CT enhanced revision of the ant genus *Zasphinctus* Wheeler (Hymenoptera, Formicidae, Dorylinae) in the Afrotropics. ZooKeys 693: 33-93. https://doi.org/10.3897/zookeys.693.13012

Hita Garcia F, Gómez K, Keller RA, Schurian B, Economo EP (2025) A never-ending story: updated 3D cyber-taxonomic revision of the ant genus *Zasphinctus* Wheeler (Hymenoptera, Formicidae, Dorylinae) for the Afrotropical region. ZooKeys 1223: 1-55. https://doi.org/10.3897/zookeys.1223.131238

Hita-Garcia F, Lieberman Z, Audisio TL, Liu C, Economo EP, Revision of the Highly Specialized Ant Genus *Discothyrea* (Hymenoptera: Formicidae) in the Afrotropics with X-Ray Microtomography and 3D Cybertaxonomy, Insect Systematics and Diversity, Volume 3, Issue 6, November 2019, 5, https://doi.org/10.1093/isd/ixz015

Richter A, Hita Garcia F, Keller RA, Billen J, Katzke J, Boudinot BE, Economo EP, Beutel RG (2021) The head anatomy of *Protanilla lini* (Hymenoptera: Formicidae: Leptanillinae), with a hypothesis of their mandibular movement, Myrmecological News, 31, pp. 85–114.

Reviewer 2 response

Reviewer #2: Well drafted and presented

A revision of the Strumigenys mnemosyne group has been nicely attempted by the authors. It would be better if images of the species are referred in key.

Many thanks for reviewing our manuscript. As suggested by the reviewer, we have now incorporated figure citations into our dichotomous key.

Reviewer 3

**PONE-D-25-13901 Reviewing conclusion 20250607 by Zhenghui Xu**

After review of the manuscript PONE-D-25-13901, I believe the three species described, S. liui sp. nov., S. marmorata sp. nov., and S. rimdahli sp. nov., are new to science. The discovery of new members obviously increased our knowledge of the rare Strumigenys mnemosyne group. The results are worth to publish on the journal. However, there are numerous errors or inappropriate expressions in the manuscript. It needs to be published after major revisions.

1. Line 6: Change “Kadoorie Biological Sciences, Building” to “Kadoorie Biological Sciences Building”.

Thank you for highlighting this. Your recommendation has been added to the manuscript at the respective location.

2. Lines 60-61: Change “singleton holotype specimens” to “singleton holotype specimen”.

Thank you for highlighting this, however, your alteration is incorrect, as we are referring to multiple holotype specimens for multiple distinct species. We have therefore retained this in the manuscript at this respective location.

3. Lines 89-91: This sentence “We provide an updated dichotomous key and distributional checklist and map of the whole S. mnenosyne group, and high-resolution images of all species, including the first for S. runa (Bolton, 2000)” used too many “and”, and the meaning of last part is not clear. I suggest the authors to use simple sentences to express clearly.

Thank you for highlighting this, we have changed this to ‘*We provide an updated dichotomous key, distributional checklist and map of the whole* S. mnenosyne *group, as well as high-resolution images of all species, including the first for* S. runa *(Bolton, 2000).’*

4. Lines 110-135: The authors used some complex abbreviation for the measurements “PrW, PetL, PetH, DPetW, DPetL, DPPetW, DDPetL”, why don’t you use the simple abbreviation “PW, PL, PH, DPW, DPL, PPW, PPL” as in Bolton (2000)? Because scientific report is trying to make thing simple, not the contrary.

Thank you for this comment. Other than PW, Bolton 2000 did not diagnose these measurement types. Nevertheless, I have simplified these acronyms by changing PrW to PW, and removed all mention of ‘Pet’ from the acronym. I have kept the D (=dorsal) to ensure that the readers know these measurements refer to dorsal measurements. The new acroynms are as follows: PrW = PW; PetL = PL; PetH = PH; DPetW = DPW; DPetL = DPL; DPPetW = DPPW; DDPetL = DPPL.

5. Lines 157-179: The authors habitually used too many “we” in the whole paragraph and seems too colloquial. I suggest the authors change the expression of sentences and reduced the use of “we” frequently.

Thank you for this comment. The word ‘we’ has been removed from the entire manuscript other than within the acknowledgement sections. The structure of various sentences has been altered to reflect the removal of this word.

6. Lines 184-185: “Hita Garcia and colleagues (2025)” is not a standard literature citation, please change it to “Hita Garcia et al. (2025)”.

Thank you for highlighting this. Your recommendation has been added to the manuscript at the respective location.

7. Line 240: This sentence “Dorsum and sides of the mesosoma declivity of propodeum” seems incorrect, please rewrite.

Thank you for highlighting this; this has been changed to ‘Dorsum and sides of the mesosoma, declivity of propodeum, waist segments and gaster all smooth and shining’.

8. Line 262: Change “1 Holotype worker” to “Holotype worker”, because holotype is a single individual.

Thank you for highlighting this. Your recommendation has been added to the manuscript at the respective location, as well as throughout the manuscript where this error occurs.

9. Lines 269-270: In “Nothing is known of the biology of S. daspleta but it is presumed to have been collected within leaf litter”, the subjective guess “but it is presumed to have been collected within leaf litter” is perilous and maybe cause a mistake. If the biological information of the species was not recorded in the original description, it is better keeping unknown until new information obtained in the future investigation.

Thank you for this recommendation, the sentence has been changed to “Nothing is known of the biology of *S. daspleta*.”

10. Line 275: Table 1 short of an end line.

Thank you for highlighting this. Your recommendation has been added to the manuscript at the respective location.

11. Line 289: Change “Physical specimens examined (n=1)” to “Physical specimen examined (n=1)”.

Thank you for highlighting this. Your recommendation has been added to the manuscript at the respective location.

12. Line 290: Change “1 Holotype worker” to “Holotype worker”; change “Baihua Lin” to “Baihua Ling”.

Thank you for highlighting this. Your recommendation has been added to the manuscript at the respective location.

13. Line 290: Change “N25.302, E.098.789” to “N25.302, E98.789”.

Thank you for highlighting this. Your recommendation has been added to the manuscript at the respective location.

14. Line 302: Change “Holotype worker (n = 1)” to “Holotype worker”, “(n = 1)” is redundant.

Thank you for highlighting this. Your recommendation has been added to the manuscript at the respective location.

15. Line 306: Change “Occiput” to “Posterior margin”, the correct name of this part is posterior margin in morphology.

Thank you for highlighting this. Your recommendation has been added to the manuscript at the respective location, as well as across the entire manuscript.

16. Line 310: Change “in full face view” to “in full-face view”.

Thank you for highlighting this. Your recommendation has been added to the manuscript at the respective location, as well as across the entire manuscript.

17. Line 314: Change “upper masticatory margin” to “Basal masticatory margin”.

Thank you for highlighting this. Your recommendation has been added to the manuscript at the respective location.

18. Lines 314-315: Change “lower half” to “apical half”.

Thank you for highlighting this. Your recommendation has been added to the manuscript at the respective location.

19. Line 319: Change “convex below eye” to “convex before eye”.

Thank you for highlighting this. Your recommendation has been added to the manuscript at the respective location.

20. Lines 323-324: The meaning of “Mesosoma undifferentiated” is not clear. It is better to change the whole sentence “ Mesosoma undifferentiated, lacking pleural sutures other than weakly impressed promesonotal suture not reaching dorsolateral margin in lateral view ” to “ Mesosoma lacking promesonotal suture on the dorsum, metanotal groove absent”.

Thank you for this suggestion. This has been changed to ‘*In dorsal view, mesosoma lacking promesonotal suture, metanotal groove absent In lateral view, promesonotal suture weakly impressed, not reaching dorsolateral margin.*’

21. Line 326: What is “Hair wheel”?

This is a structure, also known as a mesopleural gland’ is located between the anterior margin of mesopleuron and lateral posterior pronotal margin and comprises an excavation lined with setae directed inwards resembling spokes of a wheel. Find more details in Wang et al., (2021).

Reference

Wang, Chu, Chung-Chi Lin, Roberto A. Keller, and Johan Billen. The ‘hairwheels' in *Strumigenys* ants are not glandular. *Asian Myrmecology* 13 (2021).

22. Line 334: This description “Petiolar spiracle located just below node on anterolateral region of peduncle” is wrong, please check and rewrite.

Thank you for highlighting this. This has been changed to ‘*Petiolar spiracle located just below node on anterolateral region of petiole.*’ at the respective location.

23. Line 341: About pilosity, the setae in Fig.3D-F are obviously shorter and stouter than in Fig.3A-C, why? It seems the shape of setae in Fig.3D-F distortion. Similar in Fig.4-8.

Thank you for highlighting this, however, both specimens are identical, and we cannot discern clearly the differences you have highlighted. Perhaps the differences you may see are associated with the shading options used during processing.

24. Line 346: Change “apically pointed setae” to “apically acute setae”.

Thank you for highlighting this. Your recommendation has been added to the manuscript at the respective location.

25. Line 354: It is not clear whose “Tergite one”? please rewrite.

Thank you for highlighting this. This has been changed to ‘Dorsum of gastral tergite one’.

26. Line 359: Change “Mandibles and antennal segments dulled by dense microsculpture” to “Mandibles and antennal segments with dense micropuncture”. Sculpture is a general term for all kind of sculptures.

Thank you for highlighting this. Your recommendation has been added to the manuscript at the respective location.

27. Line 362: Change “Overall body reddish-orange, appendages yellow. Spongiform tissue yellow-white” to “Body reddish orange, appendages yellow. Spongiform tissue light yellow”.

Thank you for highlighting this. Your recommendation has been added to the manuscript at the respective location.

28. Line 385: Change “tolerance for higher elevations conditions” to “tolerance for higher elevation condition”.

Thank you for highlighting this. Your recommendation was changed to ‘tolerance for higher elevation conditions’ instead.

29. Line 395: Change “In full face view” to “In full-face view”.

Thank you for highlighting this. Your recommendation has been added to the manuscript at the respective location.

30. Lines 395-396: “in lateral view” is repeated.

Thank you for highlighting this. Your recommendation has been added to the manuscript at the respective location.

31. Line 397: Change “Upper masticatory margin” to “Basal masticatory margin”.

Thank you for highlighting this. Your recommendation has been added to the manuscript at the respective location.

32. Line 398: Change “lower half” to “apical half”.

Thank you for highlighting this. Your recommendation has been added to the manuscript at the respective location.

33. Line 406: Change “1 Holotype worker” to “Holotype worker”.

Thank you for highlighting this. Your recommendation has been added to the manuscript at the respective location.

34. Line 406: Change “22.26906 113.93088” to “N22.26906, E113.93088”.

Thank you for highlighting this. Given that all specimens now have specimen collection labels printed, and the importance of maintaining consistency between label and data presented in the paper, we think it will be prudent not add in N and E for both latitude and longitude respectively. Also, given that the country and location data are given for all specimens, we do not think it is too difficult for readers to understand that the latitude and longitudes given correspond to locations in the northern and eastern hemispheres. However, we will ensure we include Northings and Eastings in the future.

35. Line 409: Change “22.51211 114.0928” to “N 22.51211, E114.0928”.

Thank you for highlighting this. Please see our response to the previous comment.

36. Line 428: Change “Holotype measurements (n = 1)” to “Holotype measurements”.

Thank you for highlighting this. Your recommendation has been added to the manuscript at the respective location.

37. Line 435” Change “Occiput” to “Posterior margin”.

Thank you for highlighting this. Your recommendation has been added to the manuscript at the respective location.

38. Line 438: Change “in full face view” to “in full-face view”.

Thank you for highlighting this. Your recommendation has been added to the manuscript at the respective location.

39. Line 439: Change “occiput” to “posterior margin”.

Thank you for highlighting this. Your recommendation has been added to the manuscript at the respective location.

40. Lines 441-442: Change “upper masticatory margin” to “basal masticatory margin”, change “lower half” to “apical half”.

Thank you for highlighting this. Your recommendation has been added to the manuscript at the respective location.

41. Line 445: Change “single ommatidia” to “single ommatidium”.

Thank you for highlighting this. Your recommendation has been added to the manuscript at the respective location.

42. Line 446: Change “convex below eye” to “convex before eye”.

Thank you for highlighting this. Your recommendation has been added to the manuscript at the respective location.

43. Line 450: The meaning of “Mesosoma undifferentiated” is not clear. It is better to change the whole sentence “Mesosoma undifferentiated, lacking pleural sutures other than weakly impressed promesonotal suture not reaching dorsolateral margin in lateral view” to “Mesosoma lacking promesonotal suture on the dorsum, metanotal groove absent”.

Thank you for this suggestion. This has been changed to ‘*In dorsal view, mesosoma lacking promesonotal suture, metanotal groove absent. In lateral view, promesonotal suture weakly impressed, not reaching dorsolateral margin.*’

44. Line 459-460: Anterior peduncle of petiole is much longer in Fig. 4A than in Fig. 4E, they are the same species, why? Meanwhile, anterior margin of postpetiole weakly concave in Fig.4C, in contrast, anterior margin of postpetiole straight in Fig.4F, why? Are they really belonging to the same species?

We believe both specimens belong to the same species. All characters mentioned here are consistent across both specimens. The reason for these subtle differences noticed by the reviewer is an artifact of the rendering process, being unable to differentiate a margin (as in the concavity of the anterior petiolar margin) and is not found in the specimen itself.

45. Line 462: “kidney bean shaped” is not a good description, please describe using geometry terms.

Thank you for highlighting this. I have changed ‘*kidney bean shaped*’ to ‘*reniform*’ at the respective location.

46. Line 462: Change “postpetiolar disc conspicuously concave” to “postpetiole conspicuously concave”.

Thank you for highlighting this. The whole sentence changed to ‘*In dorsal view, postpetiole reniform, anterior margin conspicuously concave, lateral margins converging in parabolic arc posteriorly, posterior margin with concave notch medially.*’

47. Line 463: “parabolic arc” is not accurate, please rewrite.

Many thanks for highlighting this; however, we think this terminology is accurate in describing how both margins are convergent. We have therefore retained this character.

48. Lines 481-482: Change “post-petiole” to “postpetiole”, change “onepair” to “one pair”.

Thank you for highlighting this. Your recommendation has been added to the manuscript at the respective location.

49. Line 486: Change “meso- and metabasitarsi and tibiae” to “meso- and meta- basitarsi and tibiae”.

Thank you for highlighting this. Your recommendation has been added to the manuscript at the respective location.

50. Lines 490-492: Change “Core body segments reddish-brown; appendages of similar but lighter colour; antennal segments brighter compared to body, considerably more orange; spongiform tissue of typical whitish yellow colouration” to “Body color reddish brown, appendages light reddish brown, spongiform tissue light yellow”.

Thank you for this simplification, I have incorporated it into the manuscript. I have retained UK British spelling.

51. Line 495: Change “micro-sculpture” to “micro-punctures”.

Thank you for highlighting this. Your recommendation has been added to the manuscript at the respective location.

52. Lines 508-522: In this paragraph, the authors say “S. marmorata most closely resembles S. mnemosyne and S. rimdahli”. They have compared S. marmorata and S. mnemosyne carefully, but no difference mentioned between S. marmorata and S. rimdahli. Please supplement and improve.

Thank you for highlighting this. We have added a few sentences to this section to differentiate both species. The following has been added:

‘*Strumigenys marmorata* is also morphologically similar to *S. rimdahli*, however both can be readily separated by the presence of a sharp dorsolateral mesosomal margin in *S. rimdahli* (rounded in *S. marmorata*), and a convex rather than flat mesosomal dorsum in *S*. *marmorata*.’

53. Lines 528-529: Change “Strumigenys emmae, (Emery, 1890)” to “Strumigenys emmae (Emery, 1890)”.

Thank you for highlighting this. Your recommendation has been added to the manuscript at the respective location.

54. Line 537: Change “Digital images of holotype (A) lateral, (B) dorsal, (C) full face view” to “Digital images of holotype (A) head, (B) lateral, (C) dorsal”.

Thank you for highlighting this. Your recommendation has been added to the manuscript at the respective location.

55. Line 556: Change “1 Holotype worker” to “Holotype worker”.

Thank you for highlighting this. Your recommendation has been added to the manuscript at the respective location.

56. Line 560: Change “22.42007 114.1829” to “N22.42007, E114.1829”, change the similar latitude and longitude in the whole paragraph from Line 558 to Line 580, and on Lines 603-610.

Similar to the previous comments, we prefer keeping the current form for consistency.

57. Line 622: Indices of gyne: CI 75.91–131.18, which showing a huge span and seems unusual, please check and make sure.

Thank you for highlighting this issue. We have remeasured this head length of the specimen in particular and found an error. The measurements and indices for our examined gynes has been corrected following the new measurements.

58. Line 625: Change “occipital border” to “posterior border”.

Thank you for highlighting this. Your recommendation has been added to the manuscript at the respective location.

59. Lines 626-627: Change “upper masticatory margin” to “basal masticatory margin”, change “lower half” to “apical half.

Thank you for highlighting this. Your recommendation has been added to the manuscript at the respective location.

60. Line 636: Change “Occipital border” to “Posterior margin”.

Thank you for highlighting this. Your recommendation has been added to the manuscript at the respective location.

61. Lines 641: Change “mesoscutum anterior margin” to “anterior margin of mesoscutum”.

Thank you for highlighting this. Your recommendation has been added to the manuscript at the respective location.

62. Line 655: The word “subclubate” seems wrong, please check and correct.

Thank you for highlighting this. Your recommendation has been added to the manuscript at the respective location.

63. Line 659: Change “ventral petiole” to “ventral face of petiole”.

Thank you for highlighting this. Your recommendation has been added to the manuscript at the respective location.

64. Lines 659-660: Change “First gastralastral tergite long, as long as petiole and postpetiole” to “First gastral tergite long, as long as petiole and postpetiole combined”.

Thank you for highlighting this. Your recommendation has been added to the manuscript at the respective location.

65. Lines 680-688: Please make sure what is “microsculpture”, micro-puncture or micro-reticulation? Because “sculpture” is a general name for every kind of sculpture including puncture, reticulation, etc.

Thank you for highlighting this, we have changed this to ‘*Mandibular dorsum with dense micro-reticulation and non-reflective. Clypeus smooth centrally, with micro-punctures on periphery*.’

66. Line 690: Change “Overall body and appendages red-orange, spongiform tissue yellow-white” to “Body and appendages reddish orange, spongiform tissue light yellow”.

Thank you for highlighting this. Your recommendation has been added to the manuscript at the respective location.

67. Line 726: Change “1 Holotype worker” to “Holotype worker”.

Thank you for highlighting this. Your recommendation has been added to the manuscript at the respective location.

68. Line 753: Change “1 Holotype worker” to “Holotype worker”, change “19.236638 98.650739” to “N19.236638, E98.650739”.

Thank you for highlighting this. Your recommendation has been added to the manuscript at the respective location.

69. Line 769: Change “Occiput” to “Posterior margin”.

Thank you for highlighting this. Your recommendation has been added to the manuscript at the respective location.

70. Line 773: Change “occiput” to “posterior margin”.

Thank you for highlighting this. Your recommendation has been added to the manuscript at the respective location.

71. Lines 775-776: Change “upper masticatory margin” to “basal masticatory margin”, change “lower half” to “apical half”.

Thank you for highlighting this. Your recommendation has been added to the manuscript at the respective location.

72. Line 780: Change “single ommatidia” to “single ommatidium”.

Thank you for highlighting this. Your recommendation has been added to the manuscript at the respective location.

73. Line 781: Change “below eye” to “before eye”.

Thank you for highlighting this. Your recommendation has been added to the manuscript at the respective location.

74. Lines 785-786: The meaning of “Mesosoma undifferentiated” is not clear. It is better to change the whole sentence “ Mesosoma undifferentiated, lacking pleural sutures other than weakly impressed promesonotal suture not reaching dorsolateral margin in lateral view ” to “ Mesosoma lacking promesonotal suture on the dorsum, metanotal groove absent”.

Thank you for highlighting this. We have changed this section to ‘*In dorsal view, mesosoma lacking promesonotal suture, metanotal groove absent. In lateral view, promesonotal suture weakly impressed, not reaching dorsolateral margin*.’

75. Lines 880 and 802: Change “post-petiole” to “postpetiole”.

Thank you for highlighting this. Your recommendation has been added to the manuscript at the respective location as well as across the entire manuscript.

76. Line 815: Change “fourc to five” to “four to five”.

Thank you for highlighting this. Your recommendation has been added to the manuscript at the respective location.

77. Lines 824-826: Change “Core body segments reddish-brown; appendages of similar but lighter colour; antennal segments brighter compared to body, considerably more orange; spongiform tissue of typical whitish yellow colouration” to “Body color reddish brown, appendages light reddish brown, spongiform tissue whitish yellow”.

Thank you for highlighting this. Your recommendation has been added to the manuscript at the respective location.

78. Line 829: Please make sure what kind of sculpture on antennae, coxae and basitarsi.

Thank you for pointing this out, we have clarified this to *‘…and micro-punctures on all antennal segments, coxae and basitarsi.*’

79. Line 838: Change “rapidly” to “easily”.

Thank you for highlighting this. Your recommendation has been added to the manuscript at the respective location.

80. Line 843: Chang “in mnemosyne” to “in S. mnemosyne”.

Thank you for highlighting this. Your recommendation has been added to the manuscript at the respective location.

81. Line 852: Change “of the Strumigenys rimdahli sp. nov.” to “of Strumigenys rimdahli sp. nov.”.

Thank you for highlighting this. Your recommendation has been added to the manuscript at the respective location.

82. Line 853: Change “Digital images (A) lateral view, (B) dorsal view, (C) full face view” to “Digital images (A) head, (B) lateral, (C) dorsal”.

Thank you for highlighting this. Your recommendation has been added to the manuscript at the respective location.

83. Line 865: Change “4.73356 116.87757 ± 50m” to “N4.73356, E116.87757”.

Thank you for highlighting this. As these letters are not on the original data label, we have only given these in brackets only.

84. Line 875: Change “Digital images (A) lateral view, (B) dorsal view, (C) full face view” to “Digital images (A) head, (B) lateral, (C) dorsal”, meanwhile, rearrange the images of Fig.9 in the order: (A) head, (B) lateral, (C) dorsal.

Thank you for highlighting this, however we believe this rearrangement is unnecessary.

85. Line 878” Change “Fig 2G–I” to “Fig. 2G–I”.

Thank you for highlighting this. Your recommendation has been added to the manuscript at the respective location.

86. Line 884: Change “highly concave” to “deeply concave”.

Thank you for highlighting this. Your recommendation has been added to the manuscript at the respective location.

87. Line 889: Change “1 Holotype worker” to “Holotype worker".

Thank you for highlighting this. Your recommendation has been added to the manuscript at the respective location.

88. Lines 907-908: Change “Key to the Strumigenys mnemosyne group (adapted from Bolton 2000; Southeast Asia Strumigenys key)” to “Key to species of the Strumigenys mnemosyne group based on worker caste”.

Thank you for highlighting this. Your recommendation has been added to the manuscript at the respective location.

89. Line 909: Change “This key branches from couplet 84 of Bolton (2000), ending at couplet 87” to “(Adapted from the Southeast Asia Strumigenys key of Bolton, 2000, branching from couplet 84 and ending at couplet 87)”.

Thank you for highlighting this. Your recommendation has been added to the manuscript at the respective location.

90. Line 911: Because the opposite sentence of antithetical couplet 1 short of “Basigastral costulae” character, please delete “Basigastral costulae absent” here.

Thank you for highlighting this. The basigastral costulae character has been removed.

91. Line 917: Change “anterodorsally . . . . . 4” to “anterodorsally . . . . . 5”.

Thank you for highlighting this. Your recommendation has been added to the manuscript at the respective location.

92. Lines 935-939: It seems the characters the authors provided to separate the two species are hard to use. Please find more other clear characters to separate the two species.

Thank you for this comment however we would like from refraining from editing much of the key for several reasons. 1) there seems to be few characters that strongly differentiate each species, with this character likely being the being the strongest. 2) much of this key derives from Barry Bolton’s work and given his expert taxonomic skills we think that this is a good character to differentiate each species. 3) this character can be easily seen when comparing the head images of figure 2A and Figure 9C.

93. Line 950: Change “The mnemosyne group” to the mnemosyne group

Thank you for highlighting this. Your recommendation has been added to the manuscript at the respective location.

94. Line 952: Change “absence of spines” to “absence of propodeal spines”.

Thank you for highlighting this. Your recommendation has been added to the manuscript at the respective location.

95. Line 954: Change “the mnemosyne group” to “the S. mnemosyne group”.

Thank you for highlighting this. Your recommendation has been added to the manuscript at the respective location.
