# Supplementary material for: A revision of the rare Strumigenys mnemosyne (Formicidae; Myrmicinae) group using micro-CT scanning, with the description of three new species, and the virtual repair of a broken paratype
Source: PLoS One. 2025 Sep 24;20(9):e0331762. doi: 10.1371/journal.pone.0331762 (PMC12459837; doi:10.1371/journal.pone.0331762)
Supplement: Attachment — Submitted filename: Second round reviewing conclusion.docx [file pone.0331762.s003.docx]

PONE-D-25-13901 Second round reviewing conclusion 20250815 by Zhenghui Xu

After a careful read of the Response to Reviewers and the revised manuscript, I noticed that the authors have fully revised and improved the manuscript. I agree to publish the paper on the journal.
